# How Is Life After Severe COVID-19? Functional Cardiopulmonary Outcome and Quality of Life

**DOI:** 10.1016/j.chpulm.2024.100056

**Published:** 2024-04-05

**Authors:** Maurizio Bernasconi, Camelia Voinea, Luca Sardella, Alessandro Felice Chiesa, Marco Previsdomini, Andreas Perren, Claudia Gamondi, Adam Ogna

**Affiliations:** a Pulmonology Division, Ente Ospedaliero Cantonale (EOC), Bellinzona, Switzerland; b Department of Intensive Care, EOC Regional Hospital of Bellinzona, Bellinzona, Switzerland; c Palliative and Supportive Care Clinic, EOC Oncology Institute of Southern Switzerland, Bellinzona, Switzerland; d Faculty of Biomedical Sciences, University of Southern Switzerland (USI), Lugano, Switzerland

**Keywords:** ARDS, cardiopulmonary exercise testing, COVID-19, outcome, quality of life

## Abstract

**Background:**

Data on long-term outcomes of COVID-19-related ARDS are scarce and rely largely on patient-reported outcomes. This study aimed to study cardiopulmonary function combined with psychosocial sequelae in a cohort of patients with severe COVID-19 requiring ICU management and intubation, with a follow-up of 12 months.

**Research Question:**

What are the functional and psychosocial sequelae 1 year after severe COVID-19 requiring ICU management and intubation?

**Study Design and Methods:**

We studied a longitudinal cohort of 39 mechanically ventilated patients with COVID-19 from the early phase of the pandemic. Pulmonary function test results, cardiopulmonary exercise testing findings, and subjective health perception data from 6 and 12 months after ICU admission were collected.

**Results:**

Twelve months after COVID-19, 19.3% of participants showed at least moderate alteration in pulmonary function test findings, and 35.7% of participants showed a pathologically reduced effort capacity by cardiopulmonary exercise testing. A considerable impact on daily activities was reported, with only 41.7% of participants being able to resume work entirely and 71% reporting a relevant health impairment resulting from residual respiratory symptoms. The health perception scores did not correlate significantly with the measured cardiopulmonary performance or lung function.

**Interpretation:**

A persistent objective limitation in physical activity was observed in this population of unvaccinated patients from the early phase of the pandemic, studied for 12 months after recovery from COVID-19-related ARDS. Patients also reported a profound impact on functional autonomy, daily activities, professional life, and health perception. Despite being attributed primarily to residual respiratory symptoms, the observed health impairment is probably multifactorial, with physical and psychological factors playing a role. The prolonged course of the symptoms and the underlying complexity should be considered in future programs for the care of patients who have recovered from COVID-19.

**Take-home Points**

**Study Question:** What are the functional and psychosocial sequelae 1 year after severe COVID-19 requiring ICU management and intubation?

**Results:** We found a pathologically reduced effort capacity in one-third of the participants and altered PFT findings in one of five participants, as well as a profound impact of the disease on patient functional autonomy, activities of daily life, and professional life only partially related to residual respiratory limitations.

**Interpretation:** Our findings underscore the complexity of health impairment after severe COVID-19, which is probably multifactorial, with physical and psychological factors playing a role.

COVID-19, caused by SARS-CoV-2, spread rapidly across China and worldwide beginning in December 2019. With the threshold of 750 million infected people worldwide exceeded at the beginning of January 2023,^1^ the SARS-CoV-2 pandemic represented an unprecedented challenge for national health care systems. Almost all countries worldwide had to deal with a large number of patients seeking treatment with a broad panel of respiratory symptoms, from mild and self-limiting diseases to ARDS, needing prolonged mechanical ventilation in the ICU or high-dependency units.^1^ The acute phase of COVID-19 had a massive impact on hospital services. It caused almost 7 million deaths worldwide,^1^ and long-term consequences in patients recovering from acute COVID-19 represent a significant public health challenge for the near future.

Long-term consequences of COVID-19 are yet to be understood fully because of their intertwined physiopathologic features and impact on health and quality of life (QoL).^2^^,^^3^ According to a recent consensus article, the post-COVID-19 condition, also known as long COVID, occurs 3 months after a probable or confirmed infection and includes symptoms not explained by an alternative diagnosis, lasting at least 2 months, and having an impact on everyday functioning.^4^ The consequences of long COVID may involve different organs and systems. Impaired pulmonary function test (PFT) results, fatigue, exercise intolerance, and shortness of breath may affect up to 60% of patients 6 months after acute disease.^3^^,^^4^ Mental health consequences are being identified among large portions of COVID-19 survivors, with a prevalence of major depressive disorders and anxiety.^5^^,^^6^ A discrete proportion of survivors shows a delay in returning to a full working capacity, with postponements that may last up to 24 months.^2^

An increasing number of studies investigating the impact of COVID-19 on lung function failed to demonstrate an association between objective PFT results and respiratory symptoms or reported exercise intolerance.^7^, ^8^, ^9^, ^10^ These studies mainly were conducted in early convalescence phases with a short follow-up,^11^, ^12^, ^13^ and largely rely on reported exercise intolerance. Cardiopulmonary exercise testing (CPET) represents the gold standard for objective measurement of exercise capacity.^14^ To the best of our knowledge, only a few studies have performed CPET in critically ill patients with COVID-19, most with a follow-up of 6 months,^15^, ^16^, ^17^, ^18^, ^19^, ^20^, ^21^ and only one study currently is available combining objective exercise capacity with patient-reported QoL with a follow-up of 12 months.^22^

This study aimed to study functional cardiopulmonary and psychosocial sequelae in a cohort of survivors of SARS-CoV-2-induced ARDS with a follow-up of 12 months. Objective exercise capacity, pulmonary function, the impact of the disease on daily activities and professional life, and health-related QoL were investigated.

## Study Design and Methods

### Study Design and Setting

We conducted a longitudinal cohort study of patients with ARDS caused by SARS-CoV-2 needing ICU admission and invasive mechanical ventilation, discharged from Locarno Hospital between March 1, 2020, and May 31, 2020. The Hospital of Locarno belongs to the public hospitals’ network (Ente Ospedaliero Cantonale) of the Southern part of Switzerland (370,000 inhabitants). It has been entirely dedicated to the care of patients with COVID-19 since late February 2020.

### ICU Management Before Enrollment

Although patients were included in the study after ICU discharge, it is important to describe ICU management before enrolment. The decision for endotracheal intubation was made by the ICU physicians considering the presence of the following clinical points: altered mental status, risk of aspiration, severe decompensated acidosis, significant hypoxemia (Pao_2_ < 60 mm Hg or arterial oxygen saturation [SaO_2_] < 90%) despite high-flow oxygen administration with a venturi mask, and signs or symptoms of respiratory distress or tissue hypoxia. ARDS was diagnosed according to the Berlin definition.^23^ The ICU teams managed all patients according to the international best practices and applied mechanical ventilation and prone positioning according to current guidelines.^24^ All patients underwent an early rehabilitation program, beginning during the ICU stay and continued in a dedicated rehabilitation clinic after hospital discharge.

### Population and Inclusion and Exclusion Criteria

We contacted all patients meeting the inclusion criteria after discharge from the hospital and invited them to participate in the study. All participants gave written informed consent. Inclusion criteria were: (1) age ≥ 18 years, (2) SARS-CoV-2 infection confirmed by polymerase chain reaction analysis, (3) ICU admission and invasive mechanical ventilation for SARS-CoV-2-induced ARDS, and (4) hospital discharge between March 1, 2020, and May 31, 2020. Patients declining or unable to give informed consent or those with significant neurologic impairment, inability to perform the study tests, or both were excluded from the study.

After inclusion in the study, the participants were assessed 6 and 12 months after ICU admission in the hospital network outpatient pneumology units. During both visits, the participants underwent a structured medical interview, physical examination, PFTs, and CPET and completed three questionnaires. Details of the tests are reported below. We defined as the primary end point the maximum exercise capacity measured by the CPET at the 12-month visit. Maximum exercise capacity at 6 months, PFT at 6 and 12 months, and QoL at 6 and 12 months were measured as secondary end points. The research ethics board approved the study (Comitato Etico Cantonale Ticinese, Bellinzona; Identifier: 020-01933/CE3716).

### CPET and PFTs

We performed CPET by bicycle spiroergometry on a Vyntus CPX system (Vyaire), according to the current guidelines.^25^ We applied an incremental load protocol with an initial warm-up period without resistance, followed by an increase in workload by 10 to 20 W/min, depending on the patient’s physical condition and medical history, to obtain a total exercise time of 8 to 12 min. The test focused on peak oxygen uptake (VO_2_) and maximum workload and included arterial blood samples at rest and effort’s maximum. Reference values for VO_2_ were calculated according to the equation of Hansen et al.^26^

CPET was considered maximum when participants reached blood lactate of ≥ 8.0 mM, a respiratory exchange ratio (defined as exhaled carbon dioxide_2_ to VO_2_) of ≥ 1.05, or a heart rate > 80% of the maximum predicted. We considered a weight-standardized peak VO_2_ of < 80% as the cutoff to define functional limitation. Ventilatory limitation to exercise was defined when breathing reserve was < 15%, particularly when associated with increased peak Paco_2_ or high dead space ventilation. A cardiovascular limitation was retained in the presence of a reduced heart rate reserve in association with signs of cardiac or circulatory abnormalities (ECG alterations, abnormal BP, low peak pulse oxygen, and steep minute ventilation to VCO_2_ slope).^14^^,^^25^ We classified as having a peripheral (muscular) limitation the participants with functional impairment without signs of a ventilatory or cardiovascular limitation.

We used a Vyntus Body System (Vyaire) for PFTs, recording and interpreting the different measurements according to American Thoracic Society and European Respiratory Society guidelines^27^, ^28^, ^29^ and using the Global Lung Function Initiative reference values.^30^, ^31^, ^32^ During forced expiration, we recorded FEV_1_ and FVC. Static lung volumes were measured using the plethysmography method, and the diffusing capacity of the lungs for carbon monoxide (Dlco) using the single breath-hold method. We considered as clinically relevant at least moderate impairment of the PFT results, defined as FEV_1_ < 70% of predicted or Dlco < 60% predicted.^29^

### Questionnaires

We administered three questionnaires to assess health-related QoL, the functional sequelae in daily activity, and the psychological impact of the disease: the St. George Respiratory Questionnaire (SGRQ),^33^ the European Organisation for Research and Treatment of Cancer Quality of Life Questionnaire (QLQ),^34^ and the Impact of Event Scale (IES)^35^ in their validated Italian version.

The SGRQ measures health impairment related to respiratory disease and consists of 16 questions. It is suitable for patients with chronic respiratory disorders and those who have recovered from ARDS.^33^^,^^36^ Scores are calculated for three domains: (1) a symptoms component, concerned with the effect of respiratory symptoms, their frequency, and severity; (2) an activity component, concerned with activities that cause or are limited by breathlessness; and (3) an impacts component, concerned with social functioning and psychological disturbances resulting from airways disease. The total score summarizes the impact of the disease on overall health status. The mean value obtained by healthy people has been reported to be 6 points (95% CI, 5-7). Scores are expressed as a percentage of overall impairment, with 100 representing the worst possible health status and 0 indicating the best possible health status.^33^

The European Organisation for Research and Treatment of Cancer Quality of Life Questionnaire is the 30-question core questionnaire of an integrated system for assessing the health-related QoL. The European Organisation for Research and Treatment of Cancer Quality of Life Questionnaire includes five functional scales, three symptom scales, and a global health status and QoL scale ranging from 0 to 100. A high score represents a higher response level. Thus, a high score for a functional scale represents a healthy level of functioning, and a high score for the global health status and QoL represents a high QoL. Still, a high score on a symptom scale represents a high burden of symptomatology.^34^

The IES measures how a stressful event affects the participant. It consists of 15 items, each with a score ranging from 0 to 75. A higher score indicates a higher impact of the event on the participant. A score of ≥ 26 suggests that the studied event had a powerful effect on the patient. A score of ≥ 35 represents the best cutoff for a probable diagnosis of posttraumatic stress disorder (PTSD).^35^^,^^37^

### Statistical Analysis

Statistical analysis was conducted using R for Windows (R Foundation for Statistical Computing). As appropriate, we calculated descriptive statistics for the primary and secondary end points and compared groups using *t* tests or Wilcoxon rank-sum tests. Because of the small number of observations, we performed only univariable regression analysis to explore associations between baseline characteristics and outcome. We set all measures to reduce missing data to the minimum. Missing data were not replaced, and patients with missing data were excluded from the respective analysis.

### Data Availability

The data associated with the study are not publicly available, but are available from the corresponding author on reasonable request.

## Results

### Demographics and Patient Characteristics

**Figure 1:**
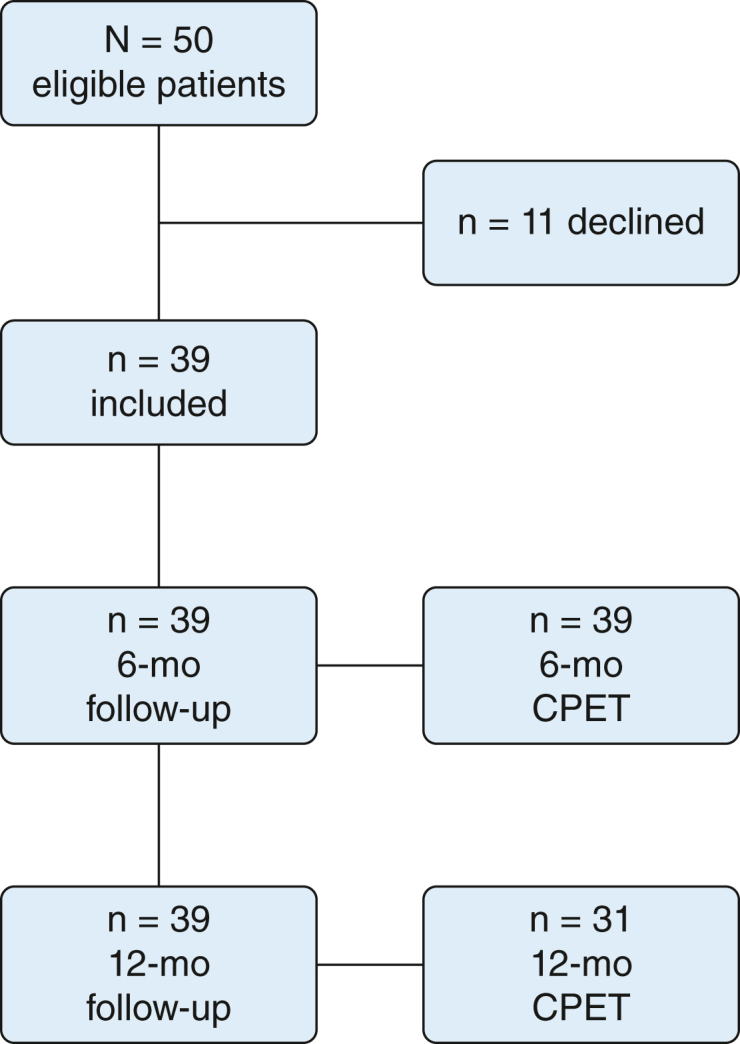
Flowchart showing patient progression through the study. CPET = cardiopulmonary exercise testing.

During the first wave of the SARS-CoV-2 pandemic from March 1, 2020, through May 31, 2020, 486 patients with COVID-19 were hospitalized in the COVID Center in Locarno; 86 patients required mechanical ventilation for COVID-19 ARDS (mean ICU stay, 30.1 ± 16.9 days). Fifty patients (58.1%) were discharged alive from the acute hospital by May 31, 2020, meeting the inclusion criteria for study enrollment. Of them, 10 patients declined to participate, and one could not attend the 6-month visit because of significant neurologic sequelae. Figure 1 reports the patient flowchart of the study.

Thirty-nine patients agreed to participate in the study and completed the 6-month evaluation. All patients took part to the 12-months follow-up visit, either on site or by phone consultation, but nine patients declined to repeat the respiratory function tests at 12 months. According to the Pao_2_ to Fio_2_ ratio, 28 patients had severe ARDS (Pao_2_ to Fio_2_ ratio, ≤ 13.3 kPa) and 11 patients had moderate ARDS (Pao_2_ to Fio_2_ ratio, 13.3-26.7 kPa). Patient characteristics and ICU management are reported in Table 1.

**Table 1:** Patient Characteristics and ICU Management

Variable	Data
Patient characteristics	
Age, y	64.1 ± 9.0
Male sex	32 (82.1)
Coexisting illness	
0	17 (43.6)
1	9 (23.1)
2	8 (20.5)
≥ 3	5 (12.8)
Diabetes	5 (12.8)
Hypertension	14 (35.9)
Obesity	19 (48.7)
COPD	2 (5.1)
Smoking history, active or previous	18 (46.2)
Hospital treatment	
Length of stay, d	35.9 ± 18.8
Tracheostomy	29 (74.3)
Timing of tracheotomy, d	12.6 ± 5.0
Mechanical ventilation duration, d	26.7 ± 18.5
Pao_2_ to Fio_2_ ratio, kPa	12.7 ± 3.7
Steroids	3 (7.7)
Antivirals	6 (15.4)
Prone positioning	39 (100)
Neuromuscular blockade	31 (79.5)

Data are presented as No. (%) or mean ± SD.

### PFTs and CPET

Thirty-one of 39 participants underwent the 12-month functional examination, 376.2 ± 11.8 days after ICU admission. Six participants (19.3%) showed at least moderate alteration of the PFT results, with impaired Dlco being the predominant finding (present in five of 31 participants). Only one patient showed a severely reduced Dlco and was the only one showing desaturation during CPET. None of the participants necessitated supplemental oxygen at rest. Ten participants (35.7%) showed a functional limitation according to CPET, with a peak VO_2_ of < 80%. Two participants (20%) demonstrated a ventilatory limitation, another two patients (20%) met the criteria for a cardiovascular limitation, and six participants (60%) showed a CPET profile suggesting a peripheral (muscular) limitation of the effort capacity. The PFT and CPET results are reported in Table 2.

**Table 2:** Pulmonary Function Test and Cardiopulmonary Exercise Test Results at the 12-Month Visit

Variable	Data
Timing of visit, d	376.2 ± 11.8
Weight, kg	93.1 ± 15.4
Height, cm	170.5 ± 4.9
BMI, kg/m^2^	32.0 ± 5.2
FEV_1_, %	100.1 ± 19.6
Vital capacity, %	97.0 ± 17.2
Total lung capacity, %^a^	88.6 ± 12.2
Dlco, %^a^	79.6 ± 19.9
Peak VO_2_, mL/kg/min	17.9 ± 3.6
Peak VO_2_, %	83.2 ± 13.1
Peak performance, W	136.1 ± 38.9
Peak performance, %	108.6 ± 24.5
RER	1.09 ± 0.08
Lowest oxygen saturation during effort, %	97.0 ± 3.9
Breathing reserve, %	41.4 ± 11.0
VD to VT	15.0 ± 3.1
VE to VCO_2_ slope	35.3 ± 8.9
Peak pulse oxygen, mL/beat	12.6 ± 3.3

Values are expressed as mean ± SD. Dlco = lung diffusing capacity for carbon monoxide; RER = respiratory exchange ratio; VD to VT = dead space ventilation; VE to VCO_2_ slope = ventilatory equivalents for CO_2_; VO_2_ = oxygen uptake; VT = tidal volume.

a One missing value (n = 30).

The results obtained at the 6-month visit showed the same proportion of respiratory functional impairment: 21.8% of participants demonstrated at least moderate alteration of pulmonary function, whereas a higher proportion of participants showed a functional limitation according to CPET (47.4%). Detailed results are available in e-Table 1. Comparing the functional evolution of participants performing both follow-up examinations, we observed a modest, albeit statistically significant, improvement in vital capacity, Dlco, and exercise capacity (e-Table 2).

### QoL and Daily Activities

Before COVID-19 hospitalization, all participants lived at home, 24 participants were professionally active, and 15 participants were retired. At 12 months, 38 participants still lived at home and one participant (2.6%) had to be housed in a nursing home. Of the 24 participants professionally active before the illness, only 10 participants (41.7%) had entirely resumed work at 12 months (seven of 39 at 6 months). Further, only 20 of the 39 participants (51.3%) reported being able to practice their previous hobbies in the same way as before.

Participants evaluated their overall QoL as good at 12 months, with a mean QLQ score of 90.66 ± 9.85 of 100 points. The SGRQ highlighted a relevant health impairment related to the disease, with 22 participants (71.0%) reporting a total SGRQ score of > 7 points. The primary health impairment was related to activity limitation by breathlessness. The IES score revealed a significant stressful impact of the disease in 13 participants (41.9%), with eight participants (25.8%) exceeding the 35-point cutoff value predictive of PTSD. Table 3 reports the questionnaire’s results at 12 months.

**Table 3:** Health Perception and Quality of Life Questionnaire Results at the 12-Month Visit

Questionnaire	Data
SGRQ domain	
Total	21.28 ± 17.97
Symptoms	15.14 ± 14.19
Activity	36.40 ± 27.54
Impact	14.17 ± 17.30
IES	22.00 ± 16.10
QLQ domain	
Global QoL	90.66 ± 9.85
Functioning	87.32 ± 12.87

Data are presented as mean ± SD. IES = Impact of Event Scale; QLQ = European Organisation for Research and Treatment of Cancer Quality of Life Questionnaire; QoL = quality of life; SGRQ = St. George Respiratory Questionnaire.

Compared with the answers reported by the same participants at the 6-month visit, the scores at the 12-month visit revealed a nonsignificant trend toward an improvement in the global QoL, with a total QLQ score increasing from 86.96 ± 11.5 of 100 points to 90.66 ± 9.8 of 100 points, and a nonsignificant trend toward worsening of the social and psychological impact component of the SGRQ score increasing from 10.83 ± 15.67 to 14.2 ± 17.30. Detailed results are available in e-Table 3.

### Correlations Among Baseline Characteristics, Functional Parameters, and Health Impairment

The comparison of participants with a functional limitation according to CPET (peak VO_2_ < 80% predicted) with those without objective limitation revealed no significant difference in baseline characteristics and hospital treatment. QoL and health perception scores also showed no significant difference between the groups. Detailed results are presented in Table 4.

**Table 4:** Comparison Between Participants With and Without Functional Limitation According to CPET Findings at the 12-Month Visit

Variable	Functional Limitation (n = 10)	No Functional Limitation (n = 21)	*P* Value
Characteristics			
Age, y	63.8 ± 9.8	63.3 ± 10.2	.895
Male sex	10 (100.0)	14 (66.7)	.066
BMI, kg/m^2^	32.0 ± 3.9	31.9 ± 6.0	.977
Diabetes	2 (20.0)	3 (14.3)	.390
Hypertension	5 (50.0)	9 (42.9)	1.00
Obesity	7 (70.0)	10 (47.6)	.280
COPD	0 (0.0)	2 (9.5)	1.00
Smoking history, active or previous	6 (60.0)	10 (47.6)	.704
Hospital treatment			
Length of stay, d	44.2 ± 28.0	31.7 ± 15.0	.213
Tracheostomy	9 (90.0)	12 (57.1)	.106
Timing of tracheotomy, d	14.6 ± 6.8	10.8 ± 3.6	.156
Mechanical ventilation duration, d	36.2 ± 29.4	23.0 ± 12.9	.204
Pao_2_ to Fio_2_ ratio, kPa	139.5 ± 31.1	133.4 ± 41.1	.665
Steroids	0 (0.0)	2 (9.5)	1.00
Antivirals	1 (10.0)	4 (19.0)	1.00
Prone positioning	10 (100.0)	21 (100.0)	1.00
Neuromuscular blockade	8 (80.0)	18 (85.7)	1.00
Lung function			
FEV_1_, %	91.5 ± 17.0	104.3 ± 19.7	.087
Vital capacity, %	88.5 ± 17.7	101.6 ± 16.0	.070
Total lung capacity, %	83.4 ± 13.0	90.4 ± 11.4	.174
Dlco, %	73.6 ± 23.0	83.4 ± 17.7	.277
CPET			
Peak VO_2_, mL/kg/min	15.6 ± 2.9	19.1 ± 3.4	.008
Peak VO_2_, %	69.2 ± 6.3	91.1 ± 8.4	< .001
Peak performance, W	116.5 ± 33.8	148.4 ± 38.2	.033
Peak performance, %	88.9 ± 20.2	121.0 ± 18.4	< .001
Breathing reserve, %	40.7 ± 15.1	41.1 ± 8.3	.937
VD to VT	16.8 ± 2.5	13.6 ± 2.8	.006
VE to VCO_2_ slope	36.1 ± 6.1	32.8 ± 5.1	.162
Peak pulse oxygen	11.6 ± 2.2	13.6 ± 3.2	.070
Questionnaire scores			
SGRQ domain			
Total	21.33 ± 20.91	20.78 ± 17.63	.944
Symptoms	15.40 ± 13.41	15.29 ± 15.27	.985
Activity	36.46 ± 29.47	36.78 ± 27.67	.978
Impact	14.01 ± 20.78	13.03 ± 16.62	.900
IES	21.90 ± 16.68	22.61 ± 17.10	.916
QLQ domain			
Global QoL	93.30 ± 8.05	89.66 ± 10.88	.327
Functioning	91.70 ± 9.00	85.96 ± 13.84	.197

Values are presented as No. (%) or mean ± SD. CPET = cardiopulmonary exercise testing; Dlco = lung diffusing capacity for carbon monoxide; IES = Impact of Event Scale; SGRQ = St. George Respiratory Questionnaire; VD = dead space; VD to VT = dead space ventilation; VE to VCO_2_ = ventilatory equivalents for CO_2_; VO_2_ = oxygen uptake; VT = tidal volume; QLQ = European Organisation for Research and Treatment of Cancer Quality of Life Questionnaire; QoL = quality of life.

Despite only two patients showing a ventilatory limitation during CPET, we found a significant correlation between the CPET performance (expressed as peak VO_2_ in % predicted) and PFT measurements, such as FEV_1_ (*r* = 0.37; *P* = .023) and Dlco (*r* = 0.50; *P* = .0015). Among baseline characteristics, only the hospital length of stay showed a significant (negative) correlation with peak VO_2_ (*r* = –0.47; *P* = .0026), whereas age and severity of ARDS (expressed as Pao_2_ to Fio_2_ ratio) showed no significant correlation. We found a significant correlation between the QLQ QoL results and both the SGRQ total score (*r* = –0.71; *P* < .001) and the IES score (*r* = –0.45; *P* = .009). No correlation was found between the QoL and health perception scores and the CPET or PFT results. Detailed correlations between peak VO_2_ and health perception scores are shown in e-Table 4.

## Discussion

Our results demonstrated an important impact on functional outcomes and patient QoL 1 year after severe COVID-19 requiring ICU management and intubation. One-third of the participants showed a pathologically reduced effort capacity by CPET, and in one of five participants, altered PFT findings of at least moderate severity persisted for 12 months.

COVID-19 showed an important impact on daily and professional activities, with a majority of participants reporting a relevant health impairment related to residual respiratory symptoms and, in particular, a limitation in activities because of breathlessness. Whereas the overall perception of QoL was preserved, a trend toward a worsening of the social and psychological impacts of the disease on perceived QoL over the study period was observed.

Our results differ from the results of the largest published study reporting 1-year outcomes in hospital survivors with COVID-19 from China,^38^ in which most patients demonstrated good physical and functional recovery during follow-up, and a large proportion of participants (88%) could return to their original work, mainly at the previous work level. The difference may be related with the greater disease severity of the present cohort. In fact, Huang et al^38^ found more symptoms, PFT results impairment, and radiographic abnormalities in the small subgroup of patients needing ICU admission (4% of the cohort, with 1% requiring invasive mechanical ventilation) when compared with participants with milder disease.

The objective pulmonary and exercise capacities we measured in our population are in line with those described in similar cohorts, reporting a relevant proportion of PFT abnormalities at 12 months, particularly Dlco impairment in 10% to 15% and an effort capacity limitation in 20% to 23% of participants.^20^, ^21^, ^22^ Similarly to the cited reports, we observed an improvement in the lung function parameters over the observation time frame.^21^^,^^22^

Persistent lung abnormalities after a severe acute lung disease requiring invasive mechanical ventilation already have been described in other coronavirus diseases, such as the 2002 severe acute respiratory syndrome epidemic and Middle East respiratory syndrome,^39^ as well as in severe ARDS of miscellaneous cause.^40^^,^^41^ In line with our observation, previous studies of survivors of ARDS found a reduced level of functional autonomy and daily life activities persisting 6 months after hospitalization and a significant reduction in health-related QoL compared with a matched population.^42^ In a recent longitudinal study, nearly one-half of previously employed survivors of ARDS were jobless 1 year after critical illness. Among those who returned to work, one-fourth became jobless during the 12-month follow-up period.^43^

In our sample, a peripheral (muscular) limitation seemed to be a leading cause of physical impairment, whereas only a few patients among those with altered lung function showed a respiratory limitation of effort capacity. These data are in line with recent studies using CPET to investigate heterogeneous cohorts of patients with COVID-19, including a few after mechanical ventilation, which suggest a peripheral component as the predominant cause of exercise limitation in patients reporting breathlessness limiting their daily activities, that may persist until 1 year after the acute disease, well beyond the early phase of convalescence after hospitalization.^15^^,^^20^^,^^21^^,^^44^, ^45^, ^46^

The peripheral limitation pattern observed during CPET could be reconducted to deconditioning or to muscular changes induced both by COVID-19 and inactivity, which may lead to mitochondrial dysfunction, myofibrillar breakdown, and reduced mitochondrial activity.^15^ These muscular changes could be explained in the current population by the exclusive selection of critically ill patients requiring invasive mechanical ventilation, neuromuscular blockade, prolonged immobilization, and ICU stay. In fact, among participants’ baseline characteristics, only the length of hospital stay showed a significant (negative) correlation with peak performance during CPET. Recent studies proposed even more complex underlying pathophysiologic characteristics: the assessment of symptomatic patients several months after recovery from mild COVID-19 by the use of invasive CPET (combining CPET with concomitant right heart catheterization) allowed the identification of heterogeneous physiologic phenotypes that could be misinterpreted as deconditioning, among which was peripheral microcirculatory dysfunction.^47^^,^^48^ A similar CPET pattern was identified in chronic fatigue syndrome.^49^

Long-lasting peripheral effort limitation occurred in this population despite an early rehabilitation program, which began during the ICU stay as soon as the patient was considered to have stable respiration, and continued in rehabilitation centers. As expected, and in line with other longitudinal studies, we observed an improvement in effort capacity over time.^21^

As previously described by other authors,^7^^,^^8^^,^^22^ we found no correlation between the QoL and health perception scores and the objective measurements of cardiopulmonary performance and PFTs. Our results align with those of Huang et al^38^ showing an increasing proportion of patients experiencing psychological consequences as more time passes after severe COVID-19.

The administration of the IES questionnaire allowed us to suspect PTSD in one of four study participants. This high incidence could be related in part to being affected by a severe form of a newly discovered disease with unknown short-term and long-term consequences and the experience of ICU treatments. Notably, PTSD, depression, and anxiety previously were reported in survivors of SARS or Middle East respiratory syndrome with similar frequencies.^50^^,^^51^ However, PTSD commonly afflicts unselected survivors of critical illness and ARDS, with a prevalence ranging from 22% to 24% in a 2-year follow-up setting.^42^

Substantial overlap with PTSD and the psychological consequences of acute COVID-19 illness could explain part of the prolonged symptoms reported as part of post-COVID-19 syndrome. Additionally, indirect effects of the disease, such as reduced social contact, loneliness, and loss of employment, could exacerbate psychological distress.

Our results must be interpreted in light of the study’s limitations. We performed a single-center study enrolling patients during the first COVID-19 pandemic phase, thus, before the availability of SARS-CoV-2 vaccines, which limits the representativeness of this cohort. Furthermore, we interpreted our results as referring to the expected normal values for a healthy population because we did not have the functional and health status of survivors of COVID-19 before acute infection, and because of the small number of participants, we were not able to adjust the results for the patient comorbidities.

## Interpretation

This study described one of the few cohorts of survivors of severe COVID-19 investigated after a 1-year follow-up combining objective physical measures coupled with subjective reports on psychological well-being and QoL. The holistic evaluation found a profound impact on patients’ functional autonomy, daily life activities, and professional life. Despite being attributed primarily to residual respiratory symptoms, the observed health impairment is probably multifactorial, with physical and psychological factors playing a role. The prolonged course of the symptoms and the underlying complexity should be considered in future programs for the care of patients after COVID-19.

## Funding/Support

The study was funded by the Southern Switzerland Respiratory League (Lega Polmonare Ticinese) and a COVID-19 dedicated research Fund of Ente Ospedaliero Cantonale.

## Financial/Nonfinancial Disclosures

None declared.

## Supplementary Data

e-Online Data
