# Supplementary material for: How Is Life After Severe COVID-19? Functional Cardiopulmonary Outcome and Quality of Life
Source: CHEST Pulm. 2024 Apr 5;2(2):100056. doi: 10.1016/j.chpulm.2024.100056 (PMC13418110; doi:10.1016/j.chpulm.2024.100056)
Supplement: e-Online Data [file mmc1.docx]

**How is life after severe COVID-19? Functional cardiopulmonary outcome and quality of life.**

**Supplemental material**

**e-Table 1:** Pulmonary Function Test and Cardiopulmonary Exercise Test (6-months visit)

| Timing of the visit (d) | 179.3 *±*13.7 |
| --- | --- |
| Weight (kg) | 86.9 *±*16.1 |
| Height (cm) | 169.5 *±*9.2 |
| BMI (kg/m^2^) | 30.1 *±*4.6 |
| FEV1 (%) | 98.9 *±*18.0 |
| Vital capacity (%) | 93.5 *±*16.5 |
| Total Lung Capacity (%) | 87.2 *±*14.1 |
| Lung Diffusion Capacity (DLCO, %) | 76.1 *±*15.8 |
| Peak VO_2_ (ml/kg/min) | 17.8 *±*3.9 |
| Peak VO_2_ (%) | 78.8 *±*14.3 |
| Peak performance (Watt) | 127.1 *±*39.4 |
| Peak performance (%) | 101.6 *±*21.5 |
| RER | 1.11 ±0.11 |
| Lowest O_2_ saturation during effort (%) | 97.3 ±3.3 |
| Breathing reserve (%) | 42.8 ±12.5 |
| VD/VT | 15.7 ±3.9 |
| VE/VCO_2_ slope | 34.2 ±6.0 |
| Peak O_2_-pulse (ml/beat) | 12.0 ±3.3 |
| Peak lactate (mmol/l)* | 6.6 ±1.9 |
| Peak PaO_2_ (kPa)* | 11.5 ±2.2 |
| Peak PaCO_2_ (kPa)* | 4.8 ±0.6 |

*Values are expressed as mean ±SD; * N=26 due to missing values*

*BMI: body mass index; FEV1: forced expiratory volume in one second; VO_2_: oxygen consumption; RER: Respiratory exchange ratio; VD: Dead space; VT: tidal volume; VE/VCO_2_: ventilatory equivalents for carbon dioxide; PaO_2_: arterial partial pressure of oxygen, PaCO_2_: arterial partial pressure of carbon dioxide.*

**e-Table 2:** comparison of Pulmonary Function Test and Cardiopulmonary Exercise Test between 6 and 12 months

|  | **6 month** | **12 month** | **p** |
| --- | --- | --- | --- |
| FEV1 (%) | 98.9 *±*18.0 | 100.1 *±*19.6 | 0.308 |
| Vital capacity (%) | 93.5 *±*16.5 | 97.0 *±*17.2 | **0.003** |
| Total Lung Capacity (%) | 87.2 *±*14.1 | 88.6 *±*12.2 | 0.790 |
| Lung Diffusion Capacity (DLCO, %) | 76.1 *±*15.2 | 79.6 *±*19.9 | **0.033** |
| Peak VO_2_ (%) | 77.3 *±*15.1 | 83.2 *±*13.1 | **0.001** |
| Peak performance (%) | 99.3 *±*21.6 | 108.6 *±*24.5 | **0.0002** |
| Breathing reserve (%) | 41.7 ±12.8 | 41.4 ±11.0 | 0.357 |
| VD/VT | 15.5 ±4.0 | 15.0 ±3.1 | 0.536 |
| VE/VCO_2_ slope | 35.0 ±6.5 | 35.3 ±8.9 | 0.759 |
| Peak O_2_-pulse (ml/beat) | 12.1 ±3.2 | 12.6 ±3.3 | 0.173 |

*Values are expressed as mean ±SD; only patients attending both study visits were analysed*

*FEV1: forced expiratory volume in one second; VO_2_: oxygen consumption, VD: Dead space; VT: tidal volume; VE/VCO_2_: ventilatory equivalents for carbon dioxide.*

**e-Table 3:** comparison of health perception and quality of life questionnaires between 6 and 12 months

|  | **6 month** | **12 month** | **p** |
| --- | --- | --- | --- |
| SGRQ: symptoms | 18.61 *±*22.39 | 15.14 *±*14.19 | 0.453 |
| SGRQ: activity | 35.78 *±*26.37 | 36.40 *±*27.54 | 0.694 |
| SGRQ: impact | 10.83 *±*15.67 | 14.17 *±*17.30 | 0.266 |
| SGRQ: total | 19.94 *±*17.85 | 21.28 *±*17.97 | 0.524 |
| IES | 23.48 *±*18.24 | 22.00 *±*16.10 | 0.804 |
| QLQ: global QoL | 86.96 *±*11.48 | 90.66 *±*9.85 | 0.104 |
| QLQ: functioning | 84.64 *±*15.47 | 87.32 *±*12.87 | 0.396 |

*Values are expressed as mean ±SD; only patients attending both study visits were analysed.*

*SGRQ: St. George's Respiratory Questionnaire; IES: Impact of Event Scale; QLQ: EORTC Quality of Life Questionnaire; QoL: quality of life*

**e-Table 4:** correlations between objective effort capacity and health perception scores

|  | **Correlation with peak VO_2_** | |
| --- | --- | --- |
|  | **r** | **p** |
| **SGRQ:** |  |  |
| - Total score | -0.026 | 0.896 |
| - Symptoms | 0.046 | 0.817 |
| - Activity | -0.084 | 0.671 |
| - Impact | 0.158 | 0.350 |
| **QLQ:** |  |  |
| - Global QoL | -0.021 | 0.917 |
| - Functioning | -0.038 | 0.847 |

*VO_2_: oxygen consumption (by Cardiopulmonary Exercise Test); SGRQ: St. George's Respiratory Questionnaire; QLQ: EORTC Quality of Life Questionnaire; QoL: quality of life*
